# The Neuromuscular Portal and Match: Working Toward a More Fair, Equitable, and Transparent Process

**DOI:** 10.1212/NE9.0000000000200086

**Published:** 2023-08-23

**Authors:** Ruple S. Laughlin, Shirlyn Adkins, Michael Baer, Sara Dehbashi, Karissa Gable, Raghav Govindarajan, Amanda C. Guidon, Kelly G. Gwathmey, Michael K. Hehir, Matthew Imperioli, Raymond S. Price, Sarada Sakamuri, Jacob Sokol, Noelle Tiongson, Ericka S. Wong, Zachary London

**Affiliations:** From the Mayo Clinic (R.S.L.); AANEM (S.A., J.S.), Rochester, MN; University of Pennsylvania (M.B., R.S.P.); Thomas Jefferson University (S.D., E.S.W.), Philadelphia, PA; Duke University (K.G.), Durham, NC; University of Missouri (R.G.), St. Louis; Department of Neurology (A.C.G.), Harvard Medical School/Massachusetts General Hospital, Boston, MA; Virginia Commonwealth University (K.G.G.), Richmond; University of Vermont (M.K.H.), Burlington; University of Connecticut (M.I.), Storrs; Department of Neurological Sciences (S.S.), Stanford University, Stanford, CA; Keck USC College of Medicine (N.T.), Los Angeles, CA; and University of Michigan (Z.L.), Ann Arbor.

## Abstract

Prior to 2021, the neuromuscular medicine fellowship application process suffered from non-standardized timelines and substantial variability. To rectify this, the American Association of Neuromuscular & Electrodiagnostic Medicine (AANEM) established a standardized application timeline and an online application portal in 2020-2021, followed by the introduction of a partial match process. In 2021-2022, AANEM launched a traditional, binding, two-way match system for fellowship positions allocation based on the Gale-Shapley stable matching algorithm. Surveys assessing perceptions of fairness in the application portal and match process were dispatched to applicants and program directors following the 2021 and 2022 recruitment cycles. In the 2020-21 cycle, 90% of program directors and 95% of applicants affirmed the standardized timeline benefited applicants. However, 57% of applicants deemed the process as unfair. All programs and most applicants (58%) favored a transition to a two-way match. The implementation of the two-way match in 2021-22 attracted participation from 97% of programs, with 80% of applicants and 95% of programs viewing the process as fair to applicants. A significant majority of both applicants (86%) and programs (94%) supported maintaining the standardized timeline and two-way match. We advocate for the universal adoption of the AANEM Match for neuromuscular fellowship recruitment and a standardized fellowship application timeline across all neurologic specialties to promote transparency, fairness, and equity for applicants.

## Introduction

The application process for fellowship training in neuromuscular medicine has changed. Before 2021, residents submitted application materials directly to the fellowship programs they were interested in without a standardized timeline. Once neuromuscular training was required to sit for the neuromuscular certification in 2013, the neuromuscular fellowship application process moved earlier and earlier into residency training. Some neurology residents submitted applications early in their postgraduate year (PGY) 3, a time when many had little or no exposure to neuromuscular or electrodiagnostic medicine.^1^ After interviews, programs were free to extend offers at any time. Some would require a response in as little as 24 hours before their offer “expired.” This rolling admission process effectively prohibited residents from exploring a range of programs that might be of interest.

## The 2020–2021 Application Process

To address these problems, the American Association of Neuromuscular & Electrodiagnostic Medicine (AANEM) committed to a standardized application and offer timeline in 2020–2021. At the heart of this process was the Neuromuscular Fellowship Application Portal, an AANEM-hosted online hub through which residents could submit application materials, communicate with programs, and receive offers, all on a standardized timeline. Application materials were released to programs on March 1, 2021. Interviews were conducted virtually from March to May and offers extended on June 1, 2021. This spring date effectively gave residents 4–6 more months to commit to neuromuscular medicine, secure letters of recommendation, bolster applications by participating in neuromuscular-specific scholarly experiences, complete their applications, and decide where to apply.

For the 2020–2021 application cycle for fellowships beginning in July 2022, the AANEM portal hosted a unique 1-sided or “partial” match process. Programs submitted rank lists, but applicants did not rank programs. Starting on June 1, 2021, the portal released offers to the top candidates in each program's rank list on the program's behalf, progressing down the programs' rank lists once offers were accepted or rejected. Fifty-two of 58 (90%) neuromuscular fellowship programs participated in the 2021 AANEM fellowship application offer system. Sixty-nine of 76 (90.8%) applicants accepted offers through the AANEM portal, filling 74.2% of the 93 positions.

The strengths of this system included the application timeline and the gap between interviews and offers.^2^ Ninety percent of program directors and 95% of applicants surveyed after offers were made felt that the standardized timeline was beneficial to applicants. However, the partial match was unpopular among both program directors and applicants.

Thirteen of 21 (62%) program directors and 7 of 38 (18%) applicants surveyed thought the offer process was unfair to programs, while 9 of 22 (41%) program directors and 10 of 38 (26%) applicants thought the offer process was unfair to applicants. Programs were incentivized to rank people who were likely to accept over their most preferred applicants. If applicants received an offer after June 1, they would know that they were not the program's top choice, and stakeholders worried that this could negatively affect the professional relationship between the fellow and their training faculty. Finally, the process was drawn out over several days, which prolonged a stressful experience for both programs and applicants. A survey of fellowship directors and applicants showed near-universal support for the standardized timeline and application portal. All program directors and most (58%) applicants favored moving to a traditional 2-way match.^2^

## The 2021–2022 Application Process

The AANEM again hosted the entire process through the Neuromuscular Application Portal in 2021–2022, using the same application timeline applied in 2020–2021. Programs posted information about their training opportunities and the number of positions sought. Applicants were allowed to view program postings and begin submitting materials on January 1, 2022, but these materials were not released to programs until March 1, 2022, after which time interviews were scheduled. Unlike the 2020–2021 recruitment cycle, a traditional, binding, 2-way match administered by the AANEM portal was used to allocate fellowship positions. The AANEM program used the Gale-Shapley stable matching algorithm similar to the algorithms used by the San Francisco and National Resident Matching Program (NRMP) match programs.^3^ All parties submitted a rank list on May 25, 2022, and a formal match took place, with all results released on June 1, 2022. Unmatched applicants and programs were free to contact each other after that time.

Fifty-six of 58 (96.5%) neuromuscular fellowship programs and 8 EMG-focused clinical neurophysiology (CNP) fellowship programs participated in the 2022 AANEM fellowship match process. Eighty-seven of 97 (89.7%) applicants matched through the AANEM portal, filling 76.3% of the 114 positions. Forty-eight of 87 (55%) matched applicants got their first choice of program, and 74 of 87 (85%) got one of their top 3 choices.

After the 2021–2022 portal match process, surveys were sent to the applicants and program directors. Similarly, for those surveyed after the 2020–2021 application cycle, 23 of 30 applicants (76.7%) and 34 of 36 program directors (94.4%) found the AANEM portal easy to use. Twenty-three of 29 applicants (79.3%) and 32 of 34 program directors (94.4%) thought the traditional 2-way match was fair to applicants, starkly contrasting to the surveyed responses from the partial match of 2020–2021. Twenty-two of 29 applicants (76.0%) and 31 of 36 program directors (86.1%) thought the traditional 2-way match process was fair to programs. Fifteen of 29 (51%) applicants felt they interviewed at more programs and 17 of 37 (45%) program directors felt they interviewed more applicants because of the 2-way match process. Most notably, 25 of 29 (86.2%) applicants and 32 of 34 (94%) program directors thought that all programs should continue to participate in a standardized application timeline and organized 2-way match. Furthermore, 26 of 29 (89%) applicants and 30 of 34 (88%) program directors felt that every neurology fellowship program should use this same timeline for applications and offers.

Among applicants who completed the survey, 16 of 30 (53%) reported that their first dedicated rotation in neuromuscular medicine or EMG during residency took place during the first half of PGY3 or later, further cementing the importance of delaying the fellowship application timeline until at least the second half of that academic year.

## Challenges Faced and Lessons Learned

For the 2 years that the AANEM has hosted an application portal, the number of neuromuscular fellowship positions has been greater than the number of applicants, leading to unfilled programs. It is unclear whether this is a long-standing trend because prior to 2020, the number of fellowship applicants was unknown. While it is possible that some of the unfilled programs could have secured trainees by circumventing the AANEM match, they would have done so to the detriment of the trainees, depriving applicants of the freedom to explore and rank all available options. Indeed, most of the applicants felt they applied to more programs and most of the program directors interviewed more applicants because of the match process; this likely expanded the diversity and depth of the applicant pool interviewed at most programs.

In 2021–2022, 2 neuromuscular programs secured commitments from applicants at their own institutions, rather than participating in the AANEM match. While residents may have personal or professional reasons to prefer to continue their fellowship training at the same institution, circumventing the AANEM match for any reason undermines the process. Some Canadian applicants applied through the portal but ended up taking positions with nonparticipating Canadian neuromuscular programs outside of the match.

An additional important but sometimes a less obvious reason to support a match process as opposed to the traditional rolling acceptance is the power dynamic at play between residents and the faculty who work at the same program. Faculty are in a position of authority and influence; they can advance or hinder resident careers, both during and after residency training. If a program offers a resident a position outside of the AANEM match, the resident may feel pressured to accept that position out of fear of retaliation, preventing them from applying to other programs that may be a better fit. In addition, without a match process, if a program has fewer positions than the number of internal applicants, the program and its own resident physicians could be in an uncomfortable position if some residents were offered slot(s) but not others. If both the program and the resident consent to participate in the AANEM match, the resident will have the prerogative to explore all of their options. While they still may choose to rank their home institution first, that decision will at least be fully informed. The downsides to a traditional 2-way match are minimal and likely outweighed by the benefits of the match process; this is supported by the overwhelming support of the 2021–2022 applicants and program directors for continuing a match in future application cycles. We concede that the match may have increased work for all parties because the number of applicants interviewed by programs and the number of programs considered by applicants likely increased. However, this increased effort was likely offset by increases in the diversity of the applicant pool at most programs.

The heterogeneity of CNP programs makes them harder to incorporate into a single-match process. For example, CNP programs can be equally divided into EMG and EEG or track based with an EMG or EEG focus; others provide training in a variety of electrophysiology studies, including a focus on intraoperative neuromonitoring. In addition, some programs adjust training curricula from year to year depending on clinical needs or trainee preference. Because of these program characteristics, it is currently not feasible to require all CNP programs that include EMG to participate in the AANEM fellowship portal or match. Unfortunately, this means that applicants may commit to a nonparticipating program before having the opportunity to explore programs participating in the match. A recent survey of 93 CNP program directors revealed that 86% were in favor of a universal timeline and 71% were in favor of a formal match process.^4^ We observed similar support for a universal timeline for all neurology fellowships among both applicants (89%) and program directors (88%). Thirty-nine percent of CNP fellowship directors favored an independent match, whereas 61% favored aligning efforts with affiliate societies.

## Next Steps

Some fellowship programs traditionally have preferred to recruit internal candidates outside of a match. However, this can create significant pressure or conflicts of interest for trainees and program directors.^5^ To promote a fair and reproducible recruitment process each year, it is in the best interest of trainees to use a match in all scenarios. It is also likely in the best interest of programs to use the AANEM match because it increases the depth of their applicant pool each year. The match also allows for both applicant and program to rank their choices by their true preference, instead of adjusting based on their assumptions of how the other parties are likely to rank. Participating programs using the AANEM portal also benefit from access to the portal, which allows them to promote their offerings and helps streamline the application process. To encourage unanimous participation, the AANEM will ban every program that solicits applications outside of the match in 2022–2023 from using the application portal in any manner during the following year's application cycle. This includes the ability to promote the program on the AANEM site, post their program listing on the AANEM portal, or communicate with applicants through the portal.

The San Francisco Match (SFMatch) and the NRMP are commercially available and have been used by other neurology fellowships. But unlike the AANEM match, these services are not free to programs and applicants and may impose restrictions on the application and match timeline that prevent optimizing the resident experience. The AANEM's match program worked well without glitches in the first iteration because it was based on the same principles as the NRMP. It will continue to make iterative improvements to the application and match process based on stakeholder feedback.

## A Unified Match

As of the 2022–2023 application cycle, there are 10 neurology fellowships that are using some kind of match, including neurocritical care, sleep, movement disorders, vascular neurology, neuro-oncology, neuromuscular, headache, epilepsy, CNP, and neuro-otology.^6,7^ Two more specialties, neuroimmunology and neuro-ophthalmology, have committed to using a match the following year.^8^ All these are using or planning to use the NRMP or SFMatch, and timelines vary with application submission as early as November PGY3 (vascular neurology, epilepsy/CNP, and neuro-oncology) and as late as July PGY4 (sleep). Even 1 subspecialty with an earlier timeline could lead to applicants making uninformed decisions.

The best way to minimize confusion and ensure parity among all applicants would be to standardize the timeline across all specialties and adopt a universal fellowship match. Internal Medicine, another field with diverse fellowships on different timelines, moved to a universal match in 2011 and found that the timeline benefited all stakeholders, with fellowship applicants benefitting the most.^9^ It provided applicants with the time they needed to make important career decisions, complete research projects, and develop relationships with advisors and would-be letter of recommendation writers. A universal neurology timeline and match would be fair, transparent, efficient, and reliable and give residents the agency to choose their fellowship with confidence.^10^

While the unified match has many merits, there are drawbacks to consider. Programs that are viewed as less desirable to applicants may find recruitment harder because they will no longer have the option to undercut competitors by compelling applicants to sign to a position earlier. It is likely that program reputation and geographic location affect the desirability of programs, so these factors may have a larger impact on recruitment through the match. If training programs in underserved communities are considered less desirable, it is possible that a universal neurology timeline and match could perpetuate health care inequity.

While the average number of interviews per applicant before the AANEM portal opened is unknown, there is a perception that a match leads applicants to apply and interview more broadly. If in-person interviews were to resume, the financial burden for applicants to attend more interviews could add to student debt and may hinder some residents from pursuing a fellowship. There is also a financial cost for the programs to host fellowship applicants and lost clinic time for faculty, both of which can add to departmental expenses. Finally, residency programs have to consider the challenge of covering hospital services and call when multiple residents are gone for fellowship interviews around the same time.

## Appendix Authors

**Table:**

Name	Location	Contribution
Ruple S. Laughlin, MD	Mayo Clinic, Rochester, MN	Study concept or design; analysis or interpretation of data
Shirlyn Adkins, JD	AANEM, Rochester, MN	Major role in the acquisition of data; analysis or interpretation of data
Michael Baer, MD	University of Pennsylvania, Philadelphia	Analysis or interpretation of data
Sara Dehbashi, MD	Thomas Jefferson University, Philadelphia, PA	Analysis or interpretation of data
Karissa Gable, MD	Duke University, Durham, NC	Study concept or design; analysis or interpretation of data
Raghav Govindarajan, MD	University of Missouri, St. Louis	Analysis or interpretation of data
Amanda C. Guidon, MD, MPH	Department of Neurology, Harvard Medical School/Massachusetts General Hospital, Boston, MA	Study concept or design; analysis or interpretation of data
Kelly G. Gwathmey, MD	Virginia Commonwealth University, Richmond	Study concept or design; analysis or interpretation of data
Michael K. Hehir, MD	University of Vermont, Burlington	Study concept or design; analysis or interpretation of data
Matthew Imperioli, MD	University of Connecticut, Storrs	Analysis or interpretation of data
Raymond S. Price, MD	University of Pennsylvania, Philadelphia	Analysis or interpretation of data
Sarada Sakamuri, MD	Department of Neurological Sciences, Stanford University, CA	Analysis or interpretation of data
Jacob Sokol	AANEM, Rochester, MN	Major role in the acquisition of data; study concept or design; and analysis or interpretation of data
Noelle Tiongson, MD	Keck USC College of Medicine, Los Angeles, CA	Analysis or interpretation of data
Ericka S. Wong, MD	Thomas Jefferson University, Philadelphia, PA	Analysis or interpretation of data
Zachary London, MD	University of Michigan, Ann Arbor	Major role in the acquisition of data; study concept or design; and analysis or interpretation of data

## Glossary

AANEM: American Association of Neuromuscular & Electrodiagnostic Medicine
CNP: clinical neurophysiology
NRMO: National Resident Matching Program
PGY: postgraduate year
SFMatch: San Francisco Match
